# Unlocking the Potential of Peach Palm (*Bactris gasipaes* Kunth) for Plant-Based Foods: A Review of Nutritional, Techno-Functional, and Bioactive Attributes

**DOI:** 10.3390/foods14234134

**Published:** 2025-12-02

**Authors:** Kartik Sharma, Suphat Phongthai, Wanli Zhang, Shusong Wu, Young Hoon Jung, Saroat Rawdkuen

**Affiliations:** 1 Unit of Innovative Food Packaging and Biomaterials, School of Agro-Industry, Mae Fah Luang University, Chiang Rai 57100, Thailand; kartik.coa@gmail.com; 2 Division of Food Science and Technology, Faculty of Agro-Industry, Chiang Mai University, Chiang Mai 50100, Thailand; su.phongthai@gmail.com; 3 School of Food Science and Engineering, Hainan University, Haikou 570228, China; zwl@hainanu.edu.cn; 4 College of Animal Science and Technology, Hunan Agricultural University, Changsha 410128, China; wush688@hunau.edu.cn; 5 School of Food Science and Biotechnology, Kyungpook National University, Daegu 41566, Republic of Korea; younghoonjung@knu.ac.kr

**Keywords:** underutilized tropical crops, functional food components, carotenoid, protein complementation, sustainable crop diversification

## Abstract

Peach palm (*Bactris gasipaes*) is an underutilized Amazonian crop with emerging relevance for plant-based food systems. Global demand for plant-based products continues to expand, reaching USD 28.38 billion in 2024, yet current formulations rely on a narrow set of ingredients with limitations in nutritional quality, functionality, sustainability, and supply-chain resilience. This review synthesizes quantitative evidence on the nutritional composition (carbohydrates 30–72% dm, protein 2–8% dm, lipids 2–14% dm), fatty acid profile, mineral density, and bioactive compounds (carotenoids up to 800 µg/g dm; phenolics 60–90 mg GAE/100 g dm) of peach palm fruit. Techno-functional properties relevant for plant-based applications, such as emulsification, water-binding, and structural contributions in bakery products and meat analogues, are critically examined, along with the effects of processing on nutrient retention and antinutrient reduction. The review also evaluates sustainability attributes and identifies key limitations, including regional cultivation, sensory constraints, and economic and technological barriers. By integrating nutritional, technological, and ecological perspectives, this work highlights the potential of peach palm as a diversified ingredient source and outlines research gaps necessary for future industrial adoption.

## 1. Introduction

Growing concerns about animal welfare, human health, and environmental sustainability have led to one of the biggest changes in the global food system, also known as the plant-based food revolution [1,2]. The plant-based food market, valued at approximately USD 28.38 billion in 2024, is projected to reach USD 176.90 billion by 2032, reflecting a compound annual growth rate of 25.7% [3]. This unprecedented growth stems from increasing consumer awareness of the environmental impact of animal agriculture, which accounts for approximately 14.5% of global greenhouse gas emissions [4]. Despite rapid growth, plant-based food systems still face challenges related to ingredient diversity, nutritional adequacy, and sensory performance [5,6,7].

**Figure 1:**
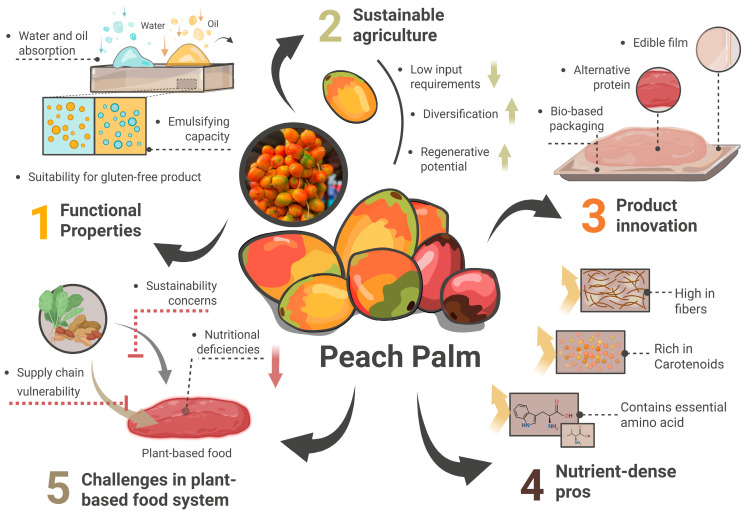
Conceptual positioning of peach palm (*Bactris gasipaes*) within modern plant-based food systems, illustrating nutritional, functional, and sustainability attributes.

Peach palm (*Bactris gasipaes* Kunth) emerges as a compelling alternative candidate to address these limitations. It is native to the Amazon basin and is widely cultivated across Central and South America, particularly in Brazil, Costa Rica, Peru, Ecuador, and Colombia. The species exhibits notable genetic diversity, with two major landraces commonly described: the macrocarpa type, characterized by larger fruits with lower oil content, and the macrocarpa type, which contains smaller, oil-rich fruits. Peach palm has long been integrated into local food cultures, where the cooked fruit is traditionally consumed as a staple carbohydrate source, processed into porridges and flours, or fermented into beverages. Beyond the fruit, the crop also supports rural economies through the production of heart of palm, an important regional and export commodity. This perennial palm species, indigenous to the Neotropical region, has sustained indigenous and rural communities for millennia through its exceptional nutritional density and year-round productivity. In contrast to annual crops with modern plant-based formulations, peach palm offers distinct advantages including continuous harvesting, minimal external inputs, and remarkable adaptability to diverse tropical environments [8]. Figure 1 illustrates the conceptual positioning of peach palm within modern plant-based food systems. The diagram summarizes key nutritional, functional, and sustainability attributes that support its potential integration into plant-based product development.

Different peach palm varieties exhibit variable nutritional advantages in terms of bioactive components. The fruit of peach palm, though low in protein content, contains all the essential amino acids and is typically rich in insoluble fiber [9]. It is well known by indigenous communities for its nutritional importance, utilizing peach palm as a primary energy source during specific seasons and incorporating it into traditional food systems to enhance dietary security [10].

The rationale for studying peach palm as a plant-based food source extends beyond its nutritional value, as climate change poses increasing risks to global food security and highlights the need for crop diversification to improve resilience and lessen environmental impacts [11,12]. Furthermore, due to its perennial growth, capacity for carbon sequestration, and comparatively low water demand, peach palm offers an environmentally advantageous alternative to conventional, resource-intensive annual crops.

Despite the growing interest in plant-based foods, there is no integrative review that synthesizes the nutritional, techno-functional, bioactive, processing, and sustainability dimensions of peach palm as a potential alternative ingredient. Existing literature is fragmented across agronomic, nutritional, and processing studies, with limited comparative evaluation and little discussion of its applicability within modern plant-based product development. Key limitations, including the lack of consolidated compositional data, inconsistent reporting of functional properties, and absence of critical assessment of sustainability and industrial relevance, remain unaddressed. This review fills these gaps by providing a comprehensive and critical synthesis that connects these previously isolated findings.

Therefore, the aim of this review is to examine peach palm’s potential in plant-based food systems through systematic analysis of its nutritional composition, processing techniques, techno-functionality, bioactivity, and applications in contemporary plant-based food development. By synthesizing current research and identifying knowledge gaps, this review aims to provide a foundation for future research directions and commercial development strategies.

## 2. Methodology of Literature Review

This review was developed using a structured literature search conducted between June and October 2025. Four major scientific databases were consulted: Scopus, Web of Science, PubMed, and Google Scholar. Search strings combined the terms “*Bactris gasipaes*” or “peach palm” with “flour,” “pulp,” “processing,” “nutritional composition,” “functional properties,” “bioactive compounds,” “heart of palm,” and “plant-based foods.” The search window covered studies published between 2020 and 2025 to capture both foundational work and recent developments in ingredient applications (Supplementary Table S1).

Studies were included if they (i) reported primary data on the nutritional composition, bioactive profile, techno-functional properties, processing effects, or food applications of peach palm; or (ii) provided relevant contextual information on plant-based systems or underutilized tropical crops. Exclusion criteria involved non-food-related uses of peach palm, studies without primary analytical data, or articles not available in English.

The identification, screening, and selection of eligible publications followed PRISMA guidelines. A simplified PRISMA flow diagram has been incorporated to illustrate the number of records identified, screened, excluded, and retained for final synthesis. A PRISMA flow diagram summarizing the identification, screening, and selection process is provided in Supplementary Figure S1. In addition, details of all records included in the systematic search and narrative synthesis are provided in Supplementary Table S1, which summarizes the full set of studies identified through the review process.

## 3. Current Plant-Based Food Market: Opportunities and Limitations

The plant-based food market is expanding rapidly, driven by sustainability concerns and the inefficiencies of animal agriculture. The Planetary Health Diet stresses the need for plant-forward food systems to meet the nutritional needs of a projected 10 billion people by 2050 [13].

Emerging plant-based trends span multiple market segments, with meat alternatives showing the most pronounced growth, followed by dairy alternatives and protein supplements [14]. Consumer motivations are linked to environmental concerns (73%), health considerations (67%), and ethical issues related to animal welfare (52%), yet sensory quality and cost remain critical barriers, with 68% of consumers identifying taste and texture as decisive factors. Current market limitations also highlight systemic vulnerabilities, including supply chain fragility; for example, pea protein prices surged by 185% during the 2021–2022 global disruptions. Nutritional adequacy concerns further persist, as plant-based diets require careful planning to prevent deficiencies in vitamin B12, iron, zinc, calcium, and omega-3 fatty acids.

The sustainability paradox of increased plant-based food consumption presents additional challenges. Although plant-based diets generally reduce environmental impact, the industrial processing required for many alternatives can increase carbon footprint and resource use, while monoculture crop expansion may contribute to biodiversity loss and soil degradation [15]. These dynamics present opportunities for diversification through the adoption of underutilized crops. Incorporating species such as peach palm could strengthen supply chain resilience, enhance nutritional profiles, and reduce environmental burdens through more sustainable cultivation practices, aligning with emerging market needs.

While peach palm offers significant potential, its adoption in plant-based food systems is limited by several factors. Its cultivation is confined to tropical regions, leading to supply fluctuations and higher transportation costs. Additionally, the flavor and texture of peach palm may not always align with consumer preferences, particularly in meat analogs and dairy alternatives. The presence of antinutritional factors like oxalates, tannins, and phytates can reduce nutrient absorption, though these can be mitigated through processing. Safety concerns also exist due to potential microbial contamination and exposure to heavy metals or pesticides from its growing environment. Lastly, high processing costs and the need for specialized equipment present economic and technological barriers that may hinder widespread commercial adoption.

Thermal processing methods such as boiling and drying are known to reduce antinutritional compounds in peach palm, including oxalates, tannins, phytates, and trypsin inhibitors, which helps improve nutrient bioavailability. Reports of allergenicity or adverse reactions are extremely limited, and although no formal Generally Recognized as Safe (GRAS) status or Novel Food approval exist for peach palm ingredients, the fruit and heart of palm have a long history of safe consumption in Latin American diets.

## 4. Nutritional Composition and Bioactive Potential: Positioning as an Alternative Source

Peach palm has two edible components: the fruit and the heart of palm. The fruit consists of the peel, pulp, and starchy mesocarp, which are the main parts explored in food science and ingredient development. This review focuses specifically on ingredients derived from the fruit pulp (cooked, dried, and milled) unless otherwise stated, as this material forms the basis of most techno-functional and product-development studies. Although peel, seed, and whole-fruit flours are occasionally referenced in research, the majority of applications relevant to plant-based foods rely on the pulp fraction. The heart of palm, while edible and commercially important, is primarily consumed as a minimally processed vegetable and is therefore not included within the scope of this ingredient-focused review.

Peach palm fruits are nutritionally dense, offering a favorable plant-based protein source. The composition varies across maturation stages and color varieties (white, yellow, orange, and red) [9]. Lipid content ranges from 2 to 14% (dm), with albino varieties reaching up to 10.8% (dm) in raw flour [16,17].

**Figure 2:**
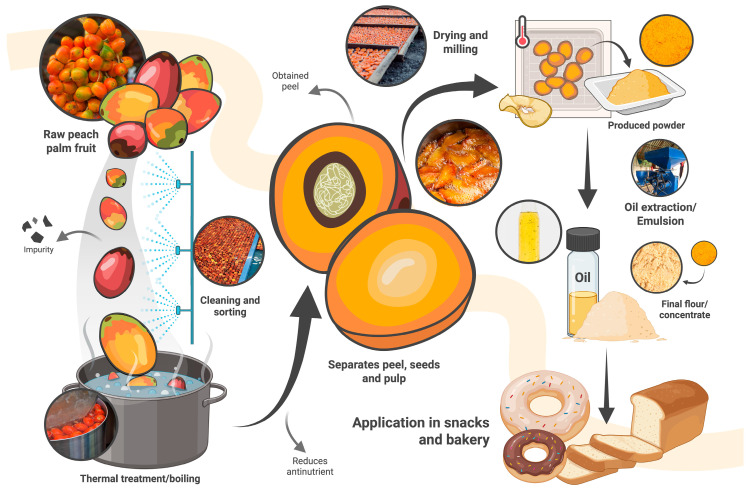
Process flow for peach palm-derived food ingredients, depicting key steps from raw fruit through cooking, drying, milling, extraction, and the production of value-added fractions like flour, oil, emulsions, etc.

Traditionally, the fruit is cooked prior to consumption to eliminate oxalate crystals in the peel and to reduce antinutritional factors such as tannins, phytates, and trypsin inhibitors [18]. This practice of cooking in water and subsequent drying to obtain flour induce several changes, including modifications in nutritional composition, cellular structure, protein denaturation, and browning, consequently affecting the color and sensorial characteristics of the fruit [16]. Figure 2 illustrates the processing steps that affect the nutritional and functional properties of peach palm products, highlighting their versatility in food innovation.

The macronutrient composition of peach palm presents a well-balanced nutritional profile, with carbohydrates comprising 30 to 72% (dry matter) (Supplementary Table S2), while starch is reported separately at 35–59 g/100 g (dm). Protein content in fresh pulp ranges from 2–8 g/100 g (dm, Table 1), depending on variety, with albino types reaching up to 7.9% (dm) in pulp and as high as 20% (dm) in flour [16,17]. In addition, it contains all essential amino acids, including notable levels of leucine and lysine reported for yellow pulp. Although the total protein and lysine/methionine content of peach palm are lower compared to soy, they are broadly comparable to rice, suggesting a need for protein complementation for optimal delivery amino acid coverage [19]. In contrast, soy protein provides a substantially higher level of most essential amino acids except methionine, and rice is characterized by low levels of lysine, with higher sulfur-containing amino acids [19]. Peach palm is also regarded as a reliable energy source compared with other Amazonian fruits like buriti (*Mauritia flexuosa*) [9,20]. Although the protein content of peach palm is lower than that of pea or soy, it is higher than many staple root crops and even some pulses, underscoring its potential as a nutritionally valuable plant-based food. Beyond macronutrients, dietary fibers are also present in substantial amounts (2–17 g/100 g) (dm), with both soluble and insoluble fractions contributing to functional and metabolic benefits. Processing of flours further concentrates both proteins and dietary fibers (as much as 20% and 17% of dm, respectively, in flour) [9,16]. The proximate composition of peach palm pulp and flours across varieties and major processing conditions highlights key nutritional differences between genotypes and processing techniques, supporting the argument for peach palm’s versatility and nutritional value in plant-based formulations (Supplementary Table S2).

Recent research also highlights the unique fatty acid composition of peach palm fruit, indicating its richness in oleic and palmitic acids (combined >75% of total fatty acids), along with moderate levels of linoleic acid and very low linolenic acids [16,19]. Compared to conventional plant oils such as soybean oil and rice bran oil, peach palm oil is higher in saturated (palmitic) and monounsaturated (oleic) fatty acids and lower in polyunsaturated fatty acids, providing a distinct profile relevant for cardiovascular health and functional food formulations [19]. Table 1 summarizes the fatty acid profile of peach palm across different color varieties.

The micronutrient density of peach palm includes a rich source of minerals including selenium, zinc, iron, copper, phosphorus, potassium, magnesium, and calcium, often at levels surpassing those of conventional plant proteins like rice, soy, or pea. For instance, cooked albino peach palm fulfils 90% of the daily requirement of magnesium for an adult and a substantial portion of dietary zinc and copper per 100 g dry matter [16,25]. Table 2 summarizes the essential macro-and trace minerals, along with recommended dietary allowance (RDA) for adults.

Overall, current evidence shows that peach palm provides a nutrient-dense profile rich in dietary fiber, carotenoids, and essential minerals. However, variability due to cultivar differences and processing methods highlights the need for standardized characterization across studies.

### 4.1. Bioactive Compounds of Peach Palm

In addition to its enriched macronutrient and micronutrient profile, peach palm is well known for its bioactive rich molecules and functional food components. Some fruit types, particularly those with orange and red fruits, along with peels (epicarps), are enriched with carotenoids, especially ß-carotene; cooked peel flour of red cultivars exceeds 800 μg/g dm [26]. This distinctive feature makes peach palm one of the richest natural sources of provitamin A, offering an important dietary supply of vitamin A for vulnerable populations [25]. These compounds contribute mechanistically through free-radical scavenging, modulation of inflammatory pathways, and enhancement of epithelial oxidative defense.

In addition, peach palm and its flours contain notable amounts of phenolic compounds (60–90 mg GAE/100 g dm), responsible for processing strong antioxidant potential. Furthermore, dietary fibers in peach palm, including resistant starch, along with polyphenol-enriched matrices, showed positive effects on colonic health, anti-inflammatory pathways, and glycemic control [20]. Recent studies indicate that dietary fibers and resistant starches present in peach palm pulps and flours can effectively modulate gut microbiota composition [26]. Such fiber-driven modulation is mechanistically linked to increased production of acetate, propionate, and butyrate.

The prebiotic effects of peach palm dietary fiber are mediated through selective fermentation by colonic bacteria, particularly *Bifidobacterium* and *Lactobacillus* species. Fermentation yields SCFA, primarily acetate, propionate, and butyrate, which activate G-protein coupled receptors (GPR41/43) and modulate AMPK-dependent metabolic pathways, thereby influencing glucose homeostasis, lipid metabolism, and epithelial barrier function [27,28,29].

Investigations into the bioaccessibility of carotenoids from peach palm fruit have shown that the high matrix density and presence of fiber can reduce micellarization efficiency during digestion, limiting absorption. Novel delivery systems, such as emulsion-based encapsulation using peach palm peel extracts, have demonstrated improved carotenoid stability and bioaccessibility during simulated gastrointestinal digestion, supporting their potential for fortified functional foods [26]. These effects align with recent findings showing that carotenoid efficacy depends strongly on micellarization efficiency, cellular uptake, and delivery-system design [30]. Generally, carotenoids from peach palm exert antioxidant effects through multiple mechanisms including direct radical scavenging, modulation of the Nrf2-ARE pathway, and suppression of NF-κB-mediated inflammatory responses. The conjugated polyene structure of β-carotene enables electron delocalization, facilitating neutralization of reactive oxygen species while preventing lipid peroxidation chain reactions [30,31]. Table 3 presents the concentrations of bioactive compounds (carotenoids, ß-carotene, lycopene, and vitamin A activity) across varieties.

In summary, peach palm contains diverse bioactive compounds with demonstrated antioxidant and functional potential. Future studies should focus on their bioaccessibility, stability, and efficacy in complex food matrices.

### 4.2. Protein Quality and Amino Acid Complementarity

Although the protein content is lower in peach palm flour, when compared to soybean or pea, its amino acid composition is relatively balanced and offers strong nutritional profile when combined with other cereals or legumes that are deficient in methionine or lysine. Complementary amino acid profiles improve protein utilization by enhancing limiting amino acids, thereby supporting more efficient biological nitrogen retention. Therefore, blending formulations can enhance the overall amino acid adequacy of plant-based diets, particularly in populations that rely heavily on plant proteins [16,20]. While the compositional data presented in the tables compare peach palm with soy and rice, a critical interpretation highlights distinct functional and nutritional roles. Soy remains substantially superior in total protein content, essential amino acid density, and gelling capacity, making it the benchmark for high-protein applications. Rice flour, in contrast, offers predictable starch-based functionality but contributes minimal carotenoids and relatively low mineral density. Peach palm occupies an intermediate position: although its protein content is lower than soy, it offers higher levels of carotenoids, dietary fiber, and minerals such as magnesium and selenium, along with a more favorable fatty acid profile than rice. These differences indicate that peach palm is best suited as a complementary ingredient that enhances micronutrient richness and functional diversity rather than replacing soy or rice in plant-based formulations. The comparison of amino acid composition of peach palm with soy and rice protein is given in Table 4. 

### 4.3. Micronutrient Advantages over Conventional Alternatives

When compared to other staple plant proteins, peach palm is not only superior in providing magnesium and selenium content but also act as natural source of provitamin A carotenoids. These nutritional attributes are hardly fulfilled by rice, soy, or pea alone, and they are particularly important in addressing the micronutrient deficiencies prevalent in plant-centric populations [16,25].

**Figure 3:**
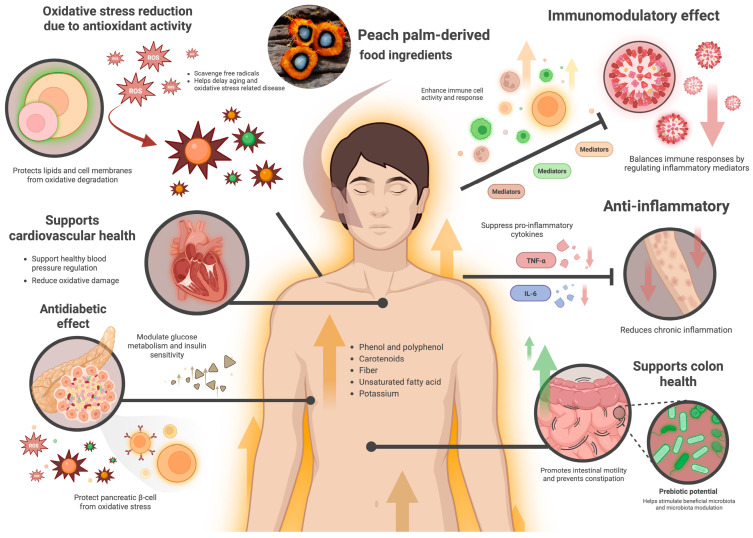
Health-promoting effects of peach palm-derived food ingredients on human physiology.

Beyond its compositional advantages, the physiological benefits of peach palm consumption have been increasingly recognized. Its unique combination of dietary fiber, unsaturated fatty acids, carotenoids, and phenolic compounds contributes to a broad range of health-promoting effects that extend from cardiovascular protection to antioxidant defense and metabolic balance (Figure 3). Importantly, most health implications discussed in this review are based on compositional data and in vitro findings, as comprehensive human clinical studies on peach palm are still lacking. These mechanisms collectively reinforce the value of peach palm not only as a nutrient-dense food source but also as a functional ingredient capable of supporting chronic disease prevention when incorporated into plant-based diets.

## 5. Functional Properties and Plant-Based Food Applications

The techno-functional behavior of peach palm can be viewed in terms of its inherent functional properties as well as the modifications introduced through processing.

### 5.1. Protein Functionality Assessment

Protein functionality is central to the application of plant-based ingredients in food formulations. It is related to attributes such as solubility, water/oil absorption, emulsifying, foaming, and gelling characteristics [35]. The emulsification capacity of peach palm proteins, though moderate compared to soy protein isolates, can be attributed to differences in protein structure and surface hydrophobicity. Soy glycinin and β-conglycinin exhibit superior interfacial properties due to their flexible molecular architecture and balanced hydrophilic–lipophilic regions. Similar structure–function relationships have been observed in other legume and cereal proteins, where thermal denaturation, pH modification, and enzymatic hydrolysis can significantly enhance surface activity and emulsion stability [36,37,38]. Comparative data on the functional properties of peach palm and related flours can be found in Supplementary Table S3. It has moderate to high water and oil absorption capacity, owing to the availability of various hydrophilic proteins and dietary fibers that aid moisture retention and softness in bakery and extruded snack products [39].

A key technological consideration for peach palm flour-based products is the oxidative stability of its lipid fraction, PUFAs such as linoleic acid. The oxidative stability of peach palm lipids is governed by the interplay between fatty acid composition, natural antioxidants (tocopherols, carotenoids), and processing-induced structural changes. The relatively high oleic acid content (>20%) provides inherent stability compared to linoleic-rich oils, while the presence of carotenoids offers synergistic protection through singlet oxygen quenching and peroxyl radical scavenging [40,41]. Recent studies on lipid oxidation kinetics in plant-based emulsions demonstrate that interfacial composition, droplet size distribution, and antioxidant localization critically determine oxidative stability during storage and digestion [40,41,42]. However, advances in extraction and processing, including ultrasound-assisted extraction and green technologies, have improved the retention of PUFAs and carotenoids in peach palm flours and emulsions, supporting better shelf life and bioactive potential in finished foods. Oxidative stability testing has demonstrated that peach palm-enriched formulations show enhanced PUFA activity and resistance to degradation over storage compared to conventional blends, strengthening their value for novel food ingredient development [17,26].

**Figure 4:**
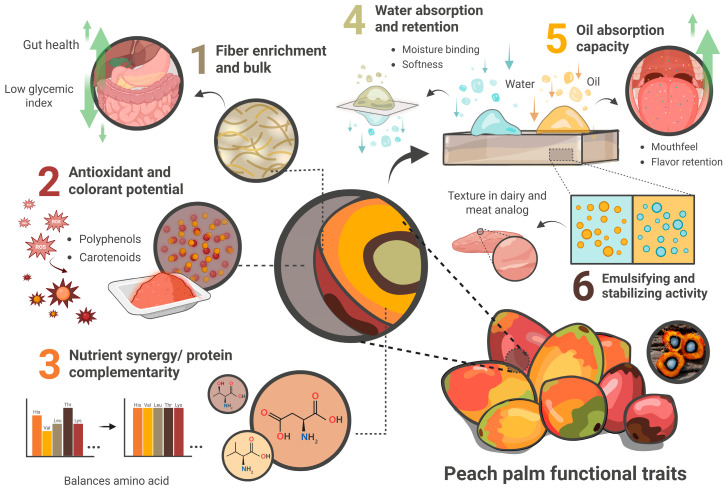
Conceptual map of the main techno-functional properties of peach palm and their relevance for different plant-based applications.

Foaming or gelling capacities are comparatively limited, likely due to lower concentrations of albumin and globulin fractions, restricting its use in applications requiring aeration or strong gel structures [9,35,39]. Emulsification and textural development during processing are further influenced by surface-active lipids and thermally induced protein–starch network formation. The overall functional versatility of peach palm is illustrated in Figure 4, which conceptually maps the main techno-functional properties of peach palm and relevance for different plant-based applications.

### 5.2. Processing Effects on Functionality

Processing methods have a substantial impact on protein functionality. For instance, the use of traditional cooking and drying methods induces protein denaturation along with enhanced water binding of peach palm, which improves digestibility while altering texture and color [9,16,43,44]. Heat treatment and extrusion further promote protein expansion and water/oil retention, though they may reduce certain heat labile bioactive compounds. Moreover, the functional performance of plant proteins is strongly influenced by factors such as extraction and drying methods, protein concentration, and processing conditions, which determine their solubility, water/oil holding capacity, emulsification, and foaming behavior [45]. Nevertheless, such treatments often enhance flavor and palatability via the Maillard reaction, a desirable effect, particularly in cereal and snack-based products [46,47]. Notably, the synergistic use of peach palm proteins with pea or soy concentrates can enhance the overall emulsification and textural performance for novel product development.

Overall, peach palm exhibits useful inherent functional properties such as water absorption, viscosity, and emulsification, while additional processing steps can further enhance or modify these characteristics.

## 6. Applications in Plant-Based Foods

The physiological relevance of peach palm ingredients reflects a combination of biological mechanisms (antioxidant activity, glycaemia modulation), chemical processes (phenolic radical neutralization and carotenoid activity), and physical functionalities (hydration capacity and matrix structuring), which collectively support their potential in plant-based formulations (Supplementary Figure S2). Peach palm four is widely used in plant-based products due to its unique composition and multifunctional properties. It is applied in gluten-free bakery goods, snacks, cereals, and meat substitutes, where it enhances nutritional value. When paired with soy or pea proteins, peach palm helps balance amino acids and improves flavor and texture [9,26,43].

In meat analogs, peach palm flour improves both nutrition and structure by enhancing moisture retention, juiciness, and firmness. Research shows that composite analogs containing peach palm fractions provide a better bite and mouthfeel while also increasing dietary fiber, micronutrients, and antioxidants [9,26]. Additionally, pairing peach palm with soy or pea proteins further strengthens gelling, water-binding properties, and amino acid balance, improving sensory quality [9,16,21,26].

Blending peach palm flour with conventional plants enhances emulsification, water binding, and gel formation, improving product stability. These blends also address amino acid limitations in cereal or legume proteins and increase oxidative stability, extending shelf life [17,19,26].

Peach palm is being developed as a protein concentrate for functional foods and sports nutrition, providing protein, fiber, carotenoids, and essential minerals. When combined with soy or rice, it offers a complete amino acid profile, making it suitable for ready-to-mix drinks and protein bars [19,25,26,43]. Its carotenoids and minerals support antioxidant capacity and recovery nutrition.

Overall, peach palm flour enhances nutrition by improving amino acid balance, antioxidants, and fiber while providing functional properties like texture, emulsification, and water retention for plant-based protein foods.

### 6.1. Dairy Alternatives and Bakery

The water absorption and gelatinization ability of peach palm flour, coupled with its mild flavor and natural pigment profile, make it suitable for formulating various dairy analogs, such as yogurt, pudding, cheese spreads, etc. The addition of lipid-rich extract derived from peach palm fruit delivers unsaturated fatty acids and natural carotenoids, such as lycopene and ß-carotene. This in turn improves color and fatty acid ratios with augmented levels of provitamin A activity. These are all desirable aspects of ‘clean-label’ dairy-free foods [19,26].

In bakery applications, peach palm flour mixed with wheat or cassava has been shown to significantly enhance crumb texture, with extended shelf life and increased consumer acceptance, even when used at levels as high as 40%. Such formulations have led to successful development of gluten-free cake and other bread mixes, offering valuable alternatives for individuals with celiac disease and broadening the range of inclusive dietary options [17,19,25,43].

### 6.2. Functional Ingredient Applications

Natural coloring and antioxidants: As discussed, peach palm peel and flour are rich sources of carotenoids (lycopene up to 10 times more in the peel in some varieties and ß-carotene); therefore, they can be used as natural bioactive food colorants and vitamin A precursors. Ultrasound-assisted and oil phase extraction have yielded pigment-rich extracts and emulsions with strong stability, making them ideal for fortifying a wide range of foods, such as baked goods, beverages, and various snack products, without the need for synthetic additives [43]. For instance, in one study [25], it was observed that an emulsion-based delivery system developed from ultrasound-assisted carotenoid extraction of peach palm peel produced physically and chemically stable emulsions for over 35 days at 30 °C. Furthermore, these emulsions retained a high percentage of carotenoids even when subjected to thermal and light stress, highlighting both their shelf stability and potential to improve the bio accessibility of lipophilic carotenoids in food fortification applications [21,25].

### 6.3. Fiber-Based Functional Ingredients

Beyond pigmentation, peach palm flour acts as a robust source of dietary fibers, both soluble and insoluble, conferring health benefits such as improved glycemic response and modulation of gut microbiota via prebiotic activity [9,21]. Fiber-rich products tend to support the design of low-glycemic, high-fiber functional foods targeted at metabolic wellness. Incorporation of peach palm into products such snacks and cereal further boost the nutritional density and functional appeal while also assisting textural engineering for processed foods. Furthermore, the presence of polyphenols and apigenin glycosides supports the claims of antioxidative and anti-inflammatory activity, expanding the spectrum of health-oriented ingredient applications [17,25,26].

### 6.4. Flavor and Texture Enhancement

The mild flavor of peach palm and its thickening capacity, along with its water-binding capacity, enhances the mouthfeel of plant-based yogurts, cheeses, and desserts. Its natural pigment composition further eliminates the need for synthetic additives [19,26]. A comparative overview of the documented food applications of peach palm-derived ingredients is summarized in Supplementary Table S4.

### 6.5. Novel Product Development Opportunities

Recent work has diversified the application of peach palm ingredients beyond traditional contexts. Details on emerging valorization strategies and novel uses of peach palm byproducts are presented in Supplementary Table S5. Briefly, stable carotenoid-rich emulsions from peach palm peel can fortify beverages, offering enhanced color, provitamin A activity, and augmented shelf life, as demonstrated in coconut-based drinks and spreads [25]. Further development of fiber and carotenoid-rich snacks and fruit pastes featuring peach palm flour offers clean-label options with added nutritional value [48]. Biodegradable packaging and antioxidant-active edible films produced from peach palm starch are being explored as sustainable alternatives for food preservation [49]. Characterization of a novel low-amylose starch from a white variety of peach palm revealed nutritional and pasting properties such as reduced retrogradation and favorable swelling or pasting behavior that make it attractive for texture modulation in gluten-free and other processed food systems [50]. The use of peach palm flour in fruit paste formulations has been demonstrated to improve consistency and fiber content while maintaining sensory acceptability, highlighting its potential in value-added pastes and snack products [51]. Resistant starch fractions from underutilized tropical fruits, including efforts relevant to peach palm, show promise as fermentable substrates for production of short-chain fatty acids, opening routes for functional food and gut-health applications [52]. Dietary fiber concentrates produced from peach palm by-products possess high water- and oil-holding capacities and gel-forming ability, supporting their incorporation into nutrition-focused formulations and ingredient blends [53]. Finally, starch structure—especially the amylose: amylopectin ratio—and clean-label modification strategies (e.g., enzymatic debranching, heat-moisture treatment, ultrasound-assisted processing) remain critical determinants of film-forming capacity, mechanical strength, and water-vapor barrier properties in biodegradable packaging applications [54,55,56]. Furthermore, the presence of low amylose fractions and resistant starch in specific peach palm varieties has supported advances in gluten-free bakery applications in the modulation of texture in processed foods [50].

Collectively, these developments demonstrate the broad potential of peach palm as a versatile ingredient that aligns with both health-focused product design and environmentally stable food systems.

## 7. Sustainability Advantages as an Alternative Crop

### 7.1. Environmental Benefits

**Figure 5:**
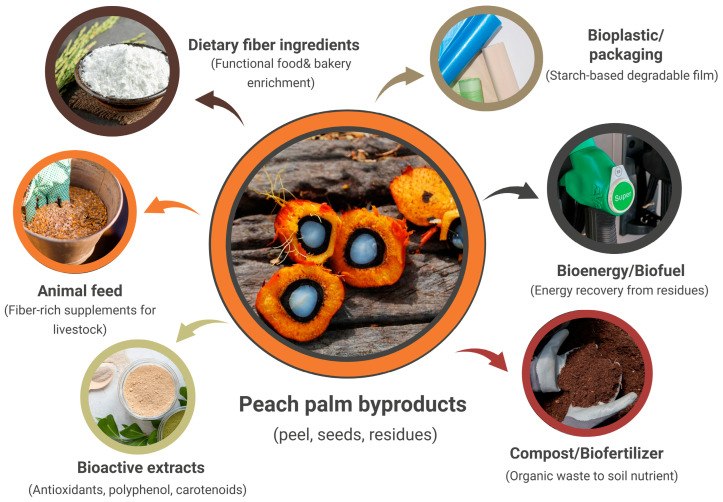
Valorization pathways of peach palm byproducts, illustrating the conversion of peels, seeds, and processing residues into value-added products such as bioactive extracts, dietary fiber concentrates, animal feed, biodegradable packaging materials, and organic fertilizers within circular bioeconomy strategies.

Peach palm stands out as an alternatively sustainable crop due to its sustainability for agroforestry systems and its ability to thrive in tropical regions with relatively low input requirements [20]. In contrast to industrial crops, it can be intercropped with native species, thereby helping to conserve biodiversity, improve soil quality, and promote carbon sequestration [20]. Its deep root system reduces soil erosion and enhances water retention, offering ecological benefits in areas vulnerable to land degradation [26]. Moreover, its byproducts such as peels and other processing residues can be utilized to produce bioactive extracts, livestock feed, and organic fertilizers, thereby valorizing the waste while mitigating the agricultural waste [26,57]. Available literature indicates that peach palm has the potential to exhibit lower environmental burdens, such as reduced land-use pressure and lower agrochemical input, when cultivated in diversified or agroforestry systems. While peach palm demonstrates favorable agronomic sustainability through perennial growth, reduced tillage requirements, and agroforestry compatibility, comprehensive life cycle assessments (LCAs) comparing environmental burdens across the full value chain remain limited. Comparative LCA studies of perennial tropical crops (e.g., breadfruit, açaí, jackfruit) indicate that integrated agroforestry systems can reduce greenhouse gas emissions by 40–60% and land-use pressure by 30–50% relative to annual commodity crops like soy and maize, suggesting similar potential for peach palm pending crop-specific quantification [58]. Despite these advantages, the current supply chain for peach palm remains limited by localized production zones and season-dependent availability, which restricts scalability for larger food manufacturing operations. Figure 5 illustrates the valorization pathways of peach palm byproducts, illustrating the conversion of peels, seeds, and processing residues into value-added products such as bioactive extracts, dietary fiber concentrates, animal feed, biodegradable packaging materials, and organic fertilizers within circular bioeconomy strategies.

### 7.2. Agricultural Sustainability

Peach palm cultivation is marked by its low agrochemical requirements, drawing from its broad genetic base and inherent pest resistance. This in turn reduces the risk of widespread disease in large-scale operations and overall environmental impact. Peach palm is compatible with agroecological approaches such as crop residue recycling and livestock integration, further advancing circular agriculture [9,20]. Recent research highlights that adopting cleaner production methods in processing substantially lessens the environmental impacts. Some facilities have been reported to eliminate more than 200 tons of waste per month in industrial operations while simultaneously creating employment opportunities in local processing communities. Its adaptability to marginal or degraded land further enhances its value, offering both income and ecosystem restoration potential [10,17,26,57].

Overall, peach palm offers strong sustainability advantages through high resource-use efficiency, adaptability, and alignment with circular bioeconomy strategies. Nonetheless, broader environmental comparisons and supply-chain assessments are required to quantify its advantages relative to major crops such as soy, pea, and wheat.

## 8. Future Outlook: Peach Palm in the Plant-Based Food Landscape

Peach palm is gaining attention in the plant-based sector due to increasing consumer interest in sustainable, nutrient-dense, and functional ingredients. Demand projections suggest its potential far exceeds its current applications. As the food industry seeks resilient, high-quality alternatives, peach palm’s unique nutritional and agronomic traits position it as a promising candidate.

Nutritionally, the fruit provides considerable amounts of dietary fibers, carotenoids, and minerals, thereby making it suitable for health-oriented and fortified formulations. Novel processing techniques now enable recovery of bioactive compounds from both the pulp and byproducts, enhancing resource efficiency and supporting zero-waste models [26]. The genetic diversity of peach palm, adaptability, and tropical origins provide opportunities for targeted breeding and regional adaptation, aligning well with the goals of regenerative agriculture and sustainable supply chains [26,50].

Advances in crop genomics and the genetic diversity of peach palm, encompassing macrocarpa and macrocarpa landraces, present opportunities for molecular breeding targeting enhanced nutritional profiles, disease resistance, and climate adaptability. Integration of multi-omics approaches (genomics, transcriptomics, metabolomics) enables identification of elite germplasm and accelerates trait selection for industrial applications. Similar strategies applied to underutilized crops have successfully identified biochemical markers linked to carotenoid accumulation, starch quality, and stress tolerance, offering a roadmap for peach palm improvement programs [59,60].

**Figure 6:**
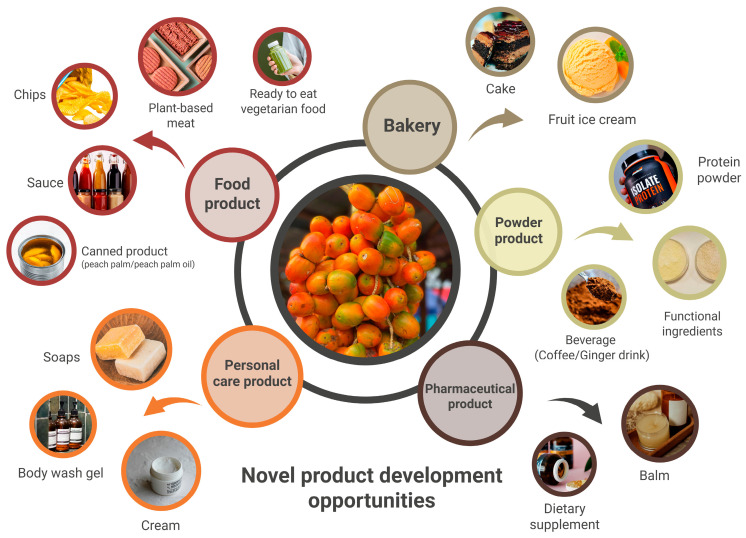
Valorization of peach palm-derived ingredients into different product categories, including plant-based foods, cosmetic formulations, and nutraceutical preparations.

Furthermore, technological development is expanding the scope of peach palm into protein blends, beverages, bioplastics, gluten-free products, cosmetic products, and biodegradable packaging, all while maintaining quality and sensory appeal for edible products. Figure 6 demonstrates some of the products (food, cosmetic, and nutraceutical products) produced via valorization of peach palm. Biodegradable packaging derived from peach palm starch further contributes to supply chain sustainability by reducing dependence on petroleum-based plastics. Importantly, localized cultivation and decentralized processing models improve economic resilience for small holders and promote community-centered value chains [25,26,49,50].

Overall, peach palm represents a highly versatile crop for advancing both sustainable food production and environmental management. Realizing its full potential will require continued innovation in breeding, scalable green technologies, and collaborative research across the supply chain.

### Industrial Viability and Technological Considerations

The industrial scalability of peach palm-derived ingredients is shaped by several practical and technological constraints. Carotenoid-rich fractions show strong potential for commercial extraction; however, yields vary widely across cultivars, and their sensitivity to heat, oxygen, and light continues to pose challenges for large-scale stabilization and formulation work [61]. Beyond carotenoids, the broader valorization of peach palm biomass faces similar barriers observed in other underutilized tropical crops, including limited access to advanced extraction technologies, inconsistent raw material supply, and the need for optimized, cost-effective processing strategies [62]. These limitations are intensified by the crop’s regional cultivation pattern, which contributes to higher transportation and processing costs when compared to globally established commodities such as soy and rice. More broadly, successful industrial integration of peach palm will require coordinated improvements in extraction efficiency, post-harvest handling, and value-chain development, consistent with trends observed for other underutilized fruit species entering sustainable food and bioprocessing markets [63].

In addition, broader market adoption will also depend on consumer familiarity with peach palm-derived ingredients, which is currently limited in many regions compared with established sources such as soy, pea, and wheat. Regulatory approval pathways for novel peach palm ingredients also vary across countries, presenting an additional consideration for future commercial development. Furthermore, sensory performance of peach palm-derived ingredients, including flavor and color perception in formulated products, remains an underexplored research area.

## 9. Conclusions

The consolidated evidence demonstrates that peach palm is a nutritionally dense, bioactive-rich, and environmentally resilient crop with strong potential for integration into plant-based food systems. Its complementary amino acid profile, valuable carotenoid and mineral composition, and favorable techno-functional properties position it as a promising ingredient rather than a direct substitute for soy or rice. However, large-scale adoption will depend on overcoming practical limitations, including regional production constraints, variability in fruit composition, the stability of carotenoid-rich fractions, and the need for cost-efficient processing infrastructure. Industrial applications appear most feasible where peach palm can enhance micronutrient density, antioxidant activity, or structural properties in blended formulations. Future research should prioritize improving post-harvest handling, optimizing scalable extraction and stabilization technologies, characterizing sensory acceptance across product categories, and developing economically viable value-chain models. Addressing these scientific and industrial gaps will be essential for positioning *Bactris gasipaes* as a strategic crop in sustainable and diversified plant-based food economies. It provides an integrative visual summary of the key nutrients, bioactive compounds, techno-functional attributes, and potential application pathways of peach palm in plant-based food systems, supporting the overall conceptual synthesis of this review.

## Figures and Tables

**Table 1:** Fatty acid composition of peach palm (*Bactris gasipaes*) pulp lipid extract by color variety.

Category	Specific Fatty Acid	Peach Palm (Red)	Peach Palm (Yellow)	Peach Palm (Green)	Peach Palm (White)	Peach Palm (Albino)	Rice Bran Oil	Soybean Oil
Medium-chain SFA (MCFSA)	Lauric (C12:0) Myristic (C14:0)	33–60 18.9–27.8	30–55 17–25	28–50 16–24	25–40 15–22	30–35 ~20	~0.2 ~0.4	~0 ~0.3
Long-chain SFA (LCFSA)	Palmitic (C16:0) Stearic (C18:0)	6–9.6 1–2	6–9 1–2	5–8 1–2	6–10 1–2	7–9 ~2	~16–20 1–3	10–12 1–5
Monounsaturated (MUFA)	Oleic (C18:1, ω-9)	21.9–24.3	20–23	18–22	20–24	19–22	39–42	22–28
Polyunsaturated (PUFA)	Linoleic (C18:2, ω-6) Linolenic (C18:3, ω-3)	4–6 0.5–1	3–5 0.5–1	3–5 0.5–1	5–7 0.7–1.2	5–7 ~1	35–37 ~1	50–54 6–8
Minor/Trace	Capric (C10:0), Arachidic (C20:0), Behenic (C22:0), etc.	<0.5 each	<0.5	<0.5	<0.5	<0.5		

Note: Values as reported in %, dry basis, mean (range) from validated sources. Ref: [9,16,19,21,22,23,24].

**Table 2:** Mineral composition of peach palm (*Bactris gasipaes*) pulp per 100 g dry matter.

Category	Mineral	Pooled Mean (mg/100 g dm)	Adult RDA/AI (per Day) (mg)
Essential (Macro)	Calcium (Ca)	194 ± 95.6	1000
	Magnesium (Mg)	196.4 ± 120.7	400
	Phosphorus (P)	163.6 ± 57.6	1000
	Potassium (K)	311	3500
	Sodium (Na)	25.1	2400
Essential (trace)	Iron (Fe)	4.41 ± 1.77	18
	Zinc (Zn)	2.56 ± 1.50	15
	Copper (Cu)	1.28 ± 0.55	2
	Manganese (Mn)	2.54 ± 1.78	2
	Selenium (Se)	553 ± 151 μg/100 g	70 μg (men), 55 μg (women)
Non-essential/contaminants	Aluminum (Al)	2.405 ± 0.046	-
	Arsenic (As)	0.555 ± 0.109	-
	Barium (Ba)	0.175 ± 0.064	-
	Cadmium (Cd)	<LOD to 0.090	-
	Chromium (Cr)	2.076 ± 0.061	-

Note: All values are per 100 g dry matter (dm); Se is in µg/100 g; LOD Limit of detection. Values represent weighted averages derived from multiple published sources; citations indicate the original data used in the calculation. Sources: [9,16,17,19].

**Table 3:** Bioactive compounds in peach palm (*Bactris gasipaes*) peel and pulp by processing and color variety (carotenoids, phenolics, vitamin A activity).

Color/Variety	Matrix	Processing	Total Carotenoids (μg/g)	Vitamin A Activity (μg RAE/g)	Total Phenolics (mg GAE/100 g)	ß-Carotene (μg/100 g Oil)	Lycopene (μg/g)
Yellow	Peel flour (dm)	Freeze-dried (raw)	43 ± 6.1	2.3 ± 0.17	33.75 ± 1.28	-	-
Red	Peel flour (dm)	Freeze-dried (raw)	481.9 ± 25.8	20.9 ± 0.54	91.39 ± 2.36	-	44.9 ± 3.8
Yellow	Peel flour (dm)	Hot air (cooked)	75 ± 7.7	3.9 ± 0.19	26.12 ± 1.34	-	1.6 ± 0.6
Red	Peel flour (dm)	Hot air (cooked)	836.5 ± 24.5	33.1 ± 0.83	83.17 ± 1.76	-	59.2 ± 2.9
Red	Pulp lipid extract	Oil phase	-	-	-	748	-
Yellow/green/white	Pulp lipid extract	Oil phase	-	-	-	32–748	-

Note: All values per 100 g dry matter. Values represent weighted averages derived from multiple published sources; citations indicate the original data used in the calculation. Sources: [17,25,26,32].

**Table 4:** Amino acid composition (mg/g protein) of peach palm (*Bactris gasipaes*), rice, and soy compared to FAO/WHO reference protein.

Amino Acid	Peach Palm	Rice	Soy	FAO/WHO Ref. (mg/g Protein)	Functional Role
Histidine	8–10	~15	~24	15	Growth, hemoglobin synthesis
Isoleucine (BCAA)	17	~32	~44	30	Muscle metabolism, energy
Lysine	31	28–31	63–67	59	Muscle protein synthesis
Leucine (BCAA)	17	~67	~75	45	Collagen, connective tissue
Methionine + Cystine (Sulfur Aas)	3–5	~30–32	~25–32	22	Methylation, glutathione synthesis
Phenylalanine + Tyrosine	44	~64	~47	38	Neurotransmitter precursors
Threonine	14–17	~35	~41	23	Mucins, immune function
Tryptophan	5–6	~11	~12	6	Serotonin, niacin precursor
Valine (BCAA)	20–23	~45	~44	39	Muscle, nitrogen balance
Total EAAs	~159 mg/g protein	240–260	270–290	277 mg/g protein	-
Non EAAs (Asp, Glu, Ala, Gly, Ser, Pro, etc.)	~240 mg/g protein	~320	~380	-	Metabolic support, flavor precursors

Sources: [22,23,24,33,34] Values represent weighted averages derived from multiple published sources; citations indicate the original data used in the calculation.

## Data Availability

No new data were created or analyzed in this study. Data sharing is not applicable to this article.

## Supplementary Materials

The following supporting information can be downloaded at: https://www.mdpi.com/article/10.3390/foods14234134/s1, Table S1: Summary of selected studies and sources included in the review; Table S2. Proximate composition of peach palm (Bactris gasipaes) pulp and flours across varieties and processing conditions; Table S3. Functional properties of peach palm (*Bactris gasipaes*) flour compared with rice and soy flour/proteins; Table S4. Application spectrum and major nutrient/functional attributes of peach palm (*Bactris gasipaes*) ingredients in food systems; Table S5. Recent novel applications and processing innovations with peach palm (*Bactris gasipaes*) ingredients in food systems; Figure S1: PRISMA flow diagram summarizing the identification, screening, and selection process; Figure S2: Integrative summary of the nutritional, bioactive, techno-functional, and application potential of peach palm (Bactris gasipaes) in plant-based food systems. References [1,2,3,4,5,6,7,8,9,10,11,12,13,14,15,16,17,18,19,20,21,22,23,24,25,26,27,28,29,30,31,32,33,34,35,36,37,38,39,40,41,42,43,44,45,46,47,48,49,50,51,52,53,54,55,56,57,58,59,60,61,62,63] are cited in the supplementary materials.

## Abbreviations

PRISMA	Preferred Reporting Items for Systematic Reviews and Meta-Analysis
GRAS	Generally Recognized as Safe
dm	dry matter
RDA	Recommended Dietary Allowance
LOD	Limit of Detection
GAE	Gallic Acid Equivalent
SCFA	Short-Chain Fatty Acids
FAO	Food and Agriculture Organization
WHO	World Health Organization
PUFA	Polyunsaturated Fatty Acids
WAC	Water Absorption Capacity
LCA	Life Cycle Assessment
