# Supplementary material for: Unlocking the Potential of Peach Palm (Bactris gasipaes Kunth) for Plant-Based Foods: A Review of Nutritional, Techno-Functional, and Bioactive Attributes
Source: Foods. 2025 Dec 2;14(23):4134. doi: 10.3390/foods14234134 (PMC12692067; doi:10.3390/foods14234134)
Supplement: Supplementary file 1 [file foods-14-04134-s001.zip › foods-3991886-supplementary.pdf]

Table S1. Summary of selected studies and sources included in the review

No.	Author (Year)	Study Type	Main Focus
1	Bryant (2022)	Review	Health and environmental sustainability of plant-based animal product alternatives
2	Kyriakopoulou et al. (2021)	Review	Functional roles of ingredients and additives in plant-based meat analogues
3	Data Bridge Market Research (2023)	Market Report	Global plant-based food market trends and forecasts
4	Steinfeld et al. (2006)	Report	Environmental impacts of livestock systems
5	Boukid (2021)	Review	Technological and market developments in plant-based meat analogues
6	Craig & Brothers (2021)	Review	Nutritional properties of non-dairy plant-based yogurt alternatives
7	McClements & Grossmann (2021)	Review	Scientific basis and construction of next-generation plant-based analogues
8	Devin et al. (2023)	Review	Orchard management and drought tolerance in fruit tree crops
9	Soares et al. (2022)	Review	Nutritional and functional properties of peach palm and its byproducts
10	González-Jaramillo et al. (2022)	Review	Traditional uses, nutritional value, and future potential of peach palm
11	Ferry & de Montalembert (2025)	Modelling Study	Crop diversification and climate vulnerability reduction
12	Mohamed Shaffril et al. (2024)	Systematic Review	Agricultural diversification as climate adaptation strategy
13	Springmann et al. (2020)	Modelling Study	Healthiness and sustainability of national dietary guidelines
14	Appiani et al. (2023)	Systematic Review	Sensory properties and consumer acceptance of plant-based analogues
15	Mehrabi et al. (2020)	Policy Analysis	Livestock policy pathways for sustainable development
16	Soares et al. (2023)	Experimental	Nutritional and technological characteristics of albino peach palm under processing
17	Costa et al. (2022)	Review	Technological potential and product applications of peach palm fruit
18	Pinheiro et al. (2022)	Experimental	Properties and industrial applications of peach palm and mammee apple seeds
19	Santos et al. (2024)	Experimental	Lipid profiles of differently coloured peach palm fruits using green extraction
20	Kramer et al. (2023)	Review	Scientific gaps and priorities for underutilized tropical species including peach palm
21	Santos et al. (2022)	Experimental	Nutritional composition and lipid profile of white peach palm variety
22	Tyagi et al. (2022)	Experimental	Amino acid and phenolic composition of rice varieties
23	Murai et al. (2024)	Experimental	Sugar and amino acid variability in U.S. soybeans and autoclaving effects
24	Dhungana et al. (2021)	Experimental	Nutritional and phytochemical variation in colored soybeans
25	Martínez-Girón et al. (2024)	Experimental	Carotenoid extraction and emulsion applications of peach palm peel
26	Spacki et al. (2022)	Review	Comprehensive valorization and uses of peach palm
27	Vinelli et al. (2022)	Systematic Review	Effects of dietary fibers on short-chain fatty acids and gut microbiota in healthy adults
28	Facchin et al. (2024)	Review	Short-chain fatty acids in human health: metabolic pathways and therapeutic implications

No.	Author (Year)	Study Type	Main Focus
29	Kok et al. (2023)	Review	Predicting personalized responses to dietary fiber interventions and gut microbiome modulation
30	Molteni et al. (2022)	Review	Improving carotenoid bio accessibility and bioavailability through nanostructured delivery systems
31	Crupi et al. (2023)	Review	Beneficial effects of carotenoids on consumer health and well-being
32	Lima et al. (2021)	Experimental	Techno-functional properties of fish byproduct protein hydrolysate
33	Xiong et al. (2023)	Experimental	Key metabolites associated with rice quality
34	Farooq et al. (2023)	Experimental	Effects of salt stress on amino acid profiles in rice
35	Sim et al. (2021)	Review	Technological roadmap for future plant protein development
36	Wang et al. (2024)	Review	Functional performance of diverse plant proteins
37	Tang et al. (2024)	Review	Recent progress in plant-based proteins: extraction, modification, and food applications
38	Chandran et al. (2023)	Review	Sustainable plant protein: sources, extraction techniques, and utilization
39	Bernardi et al. (2024)	Experimental	Nutritional quality and snack application of peach palm flour
40	Beltrame et al. (2023)	Experimental	Lipid structure influences digestion and oxidation of omega-3 fatty acids
41	Islam et al. (2023)	Review	Functional roles and tools for improving oxidative stability of polyunsaturated fatty acids
42	Aslam et al. (2023)	Review	Lipid oxidation in food emulsions: role of interfacial area
43	Silva Ribeiro et al. (2021)	Experimental	Peach palm flour: production, hygroscopic behavior, and cookie application
44	Martínez-Girón et al. (2022)	Experimental	Physicochemical and techno-functional properties of peach palm peel flour
45	Ma et al. (2022)	Review	Functional performance of plant proteins (duplicate entry - same as #36)
46	Sharma et al. (2024)	Experimental	Debittering of salmon protein hydrolysate via Maillard reaction
47	Sharma et al. (2025)	Experimental	Cookies fortified with debittered salmon hydrolysate
48	Martínez-Girón et al. (2025)	Experimental	Carotenoid-enriched coconut beverage formulated with peach palm by-products
49	Costa et al. (2023)	Experimental	Biodegradable film development from peach palm by-products
50	Rosário et al. (2025)	Experimental	Functional and pasting properties of low-amylose starch from white peach palm
51	Samaniego-Puertas et al. (2025)	Experimental	Use of peach palm flour in papaya paste formulation
52	Kembabazi et al. (2025)	Review	Resistant starch from underutilized fruits for fermentation and SCFA production
53	Giombelli et al. (2023)	Experimental	Dietary fiber concentrates from peach palm by-products
54	Rashwan et al. (2024)	Review	Plant starch extraction, modification, and green applications
55	Compart et al. (2023)	Review	Customizing starch properties: modifications and applications
56	Xie et al. (2025)	Review	Comprehensive review of starch structure, properties, and applications in food preservation
57	Varella et al. (2022)	Case Study	Cleaner production practices in peach palm heart processing
58	Quevedo-Cascante et al. (2023)	Systematic Review	Life cycle assessment capture of environmental impacts in agroforestry systems
59	Mahmood et al. (2022)	Review	Multi-omics revolution to promote plant breeding efficiency

No.	Author (Year)	Study Type	Main Focus
60	Kumar et al. (2024)	Review	Advances in genomic tools for plant breeding: molecular markers, genomic selection, and genome editing
61	Bastos et al. (2025)	Review	New strategies for antioxidant extraction from fruits and vegetables
62	Hasan et al. (2024)	Review	Trends and challenges of fruit by-products utilization: sustainable valorization approach
63	Marappan et al. (2025)	Review	Underutilized fruit crops for nutritional security and economic growth

Table S2. Proximate composition of peach palm (*Bactris gasipaes*) pulp and flours across varieties and processing conditions.

Variety/color	Processing	Moisture (%) (wb)	Protein (%) (dm)	Lipids (%) (dm)	Total carbohydrates (%) (dm)	Starch (%)	Fiber (%) (dm)	Ash (%) (dm)
Macrocarp (red-green)	Fresh pulp	44-68.5	2.4-6.9	1.9-23	-	44.32-59.50 (BNR)	0.7-9.3	0.5-1.3
Microcarp	Fresh pulp	62	2-4	6-14	-	35-54 (BNR)	3-6	0.7-2.
Mesocarp (yellow-green)	Fresh pulp	64	2-4	3-10	30-40	-	3-6	0.7-2.4
Albino (white)	Raw pulp	61	7.9	10.2	72.1	-	13.6	9.7
Albino (white)	Cooked pulp	70.5	7.6	8.6	70.	-	12.8	13.9
Albino (white)	Raw flour (55°C dried)	9.7	17	10.8	69.9	-	16.9	2.2
Albino (white)	Cooked flour (55°C dried)	5.8	20	7.9	69.6	-	8.2	2.1

Source: [9; 16; 17]

Table S3. Functional properties of peach palm (*Bactris gasipaes*) flour compared with rice and soy flour/proteins.

Functional property	Peach palm flour (peel)	Rice protein/flour	Soy protein/isolate
Water absorption (g/g)	5.3-7.1; high	2.5-3.5; moderate	2.7-3; moderate
Oil absorption (g/g)	6.2; high	1.6-2.2; medium	2-2.3; medium
Emulsifying activity (%)	56.8; moderate/high	38-45; low	45-64; high
Emulsifying stability (%)	50.3; moderate	28-35; low	60-70; high
Foaming capacity (%)	None/very low	10-30 (low)	Upto 70 (high)
Gelling capacity (%)	None (no gelling)	Poor (>14)	Strong (8-11)
Processing effects	Cooking/extrusion-increases absorption, digestibility; reduces some bioactives	Cooking-improves solubility, digestibility	Extrusion-enhances gelling, emulsification
Best applications	Snacks, bakery, high fiber blends	Gluten-free, hypoallergenic foods	Meat, fairy, textured products

Source: [9; 16; 39; 43; 44; 45]
Values represent weighted averages derived from multiple published sources; citations indicate the original data used in the calculation

Table S4. Application spectrum and major nutrient/functional attributes of peach palm (*Bactris gasipaes*) ingredients in food systems.

Application Area	Product	Peach Palm Role/Ingredient	Key Functional Contributions	Major bioactive or nutrient	Highlights	Reference
Meat analogues	Patties, nuggets, hybrid burgers	Flour, blends, fiber, oil	Texture, juiciness, water-oil binding, color, micronutrient enrichment	Carotenoids, unsat. FAs, dietary fiber, Mg, P	Improves moisture, color and micronutrient content, synergizes with soy/pea for firmer gels	[9,16,19,26]
Dairy alternatives	Yogurt, pudding, cheese, beverages	Flour/extract/oil	Texturing, thickening, emulsification, color, fiber enrichment	Resistant starch, β-carotene, phenolics, fiber	Develops firm gel-like textures, improves creamy mouthfeel & color, supports allergen-free/vegan blends	[16,19,25]
Bakery products	Cookies, cakes, bread, pasta, extrudates	Whole/peel flour, blends	Texture softening, water holding, fiber/fat enrichment, natural coloring	Fiber, starch, carotenoids, minerals, tocopherols	Up to 40% inclusion: softens crumb, increases fiber, offers yellow-orange hue, extends shelf life, positive sensory tests	[16,17,19,25,26,43]
Functional snacks	Extruded cereals, breakfast bars	Flour, byproduct blend	Fiber enrichment, prebiotic potential, antioxidant activity, color	Soluble/insoluble fiber, polyphenols, carotenoids	Significant boost in fiber/caro. content; good expansion and physical properties; consumer buy intent	[16,25,26,43]
Protein/fiber supplements	Powdered blends, ready-to-mix	Protein fraction, flour	Protein & amino acid diversity, fiber, micronutrient, bioactive boosts	Lysine, methionine (moderate), β-carotene, K, P, Mg	Synergistic blends cover EAA profile gaps, boost antioxidant value, improve GI and metabolic health	[16,26,27]
Natural colorant	Beverage, bakery, spreads, snacks	Color extract, peel flour	β-carotene coloring, vitamin A, oxidative stability, clean label	β-carotene, lycopene, apigenins	Stable emulsions, 35+ days shelf, retained color at temp/light stress, replaces synthetic dyes	[19,25]

Application Area	Product	Peach Palm Role/Ingredient	Key Functional Contributions	Major bioactive or nutrient	Highlights	Reference
Health-oriented & functional foods	GF and fiber-enriched	Whole/peel flour, byproduct	Prebiotic, glycemic moderation, cholesterol reduction	Fiber, apigenins, feruloyl quinic acids	High satiety, modulates gut flora, lower glycemic index, anti-inflammatory	[16,19,25, 26, 32]
Novel/industrial	Bioactive delivery (emulsions/encapsulation), packaging, animal feed	Extracts, starch, byproducts	Stability, controlled release, enhanced bioaccessibility, sustainable material	Starch, fiber, unsaturated fats, carotenoids	Food-safe films, shelf-stable emulsion-based bioactives, proven micronutrient delivery	[17,25,26]

Table S5. Recent novel applications and processing innovations with peach palm (*Bactris gasipaes*) ingredients in food system.

**Product**	**Peach Palm Ingredient**	**Application Area**	**Processing**	**Functional Benefit**	**Outcomes**	**Reference**
Carotenoid emulsion for beverage fortification	Red epicarp oil/pigment	Dairy alternatives, functional drinks	Ultrasound-assisted extraction, emulsion technology	Enhanced provitamin A, improved color, shelf stability	Coconut beverages enriched with β-carotene, up to 91% retention after storage	[25,48]
Papaya–peach palm fruit paste snack	Peach palm flour	Clean-label snacks	Direct blending/thermal processing	High dietary fiber and carotenoid content	New fruit paste with elevated nutrition and sustainability	[51]
Edible/biodegradable antioxidant films	Whole flour/starch	Functional packaging	Film casting/antioxidant activity analysis	Antioxidant-active, opaque, edible	Protects food and supports sustainability goals	[49]
Gluten-free, low-amylose starch ingredient	Starch from white variety	Gluten-free baking, structure modifier	Isolation, physicochemical analysis	Gluten-free, improved textural attributes	Basis for new GF formulations with unique properties	[50,52]
Dietary fiber and antioxidant-rich concentrates	Processed by-products	High-fiber/antioxidant enrichment	Dietary fiber extraction and functional characterization	Elevated fiber, strong antioxidant capacity	Substrate for metabolic wellness foods and functional beverages	[53]
Microencapsulated carotenoids and oil ingredients	Peel and oil extracts	Dairy, bakery, and beverage enrichment	Microencapsulation, oxidative stability testing	Prolonged carotenoid stability and bioavailability	Suitable for high-temperature/long-shelf-life foods	[25]

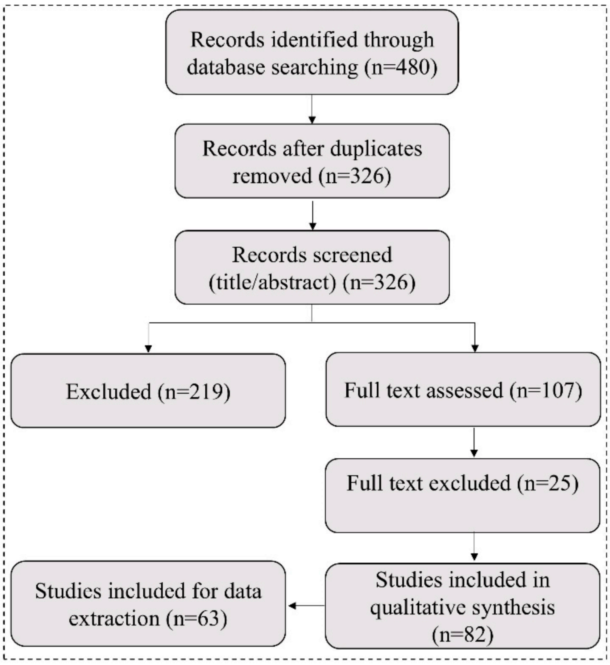

Figure S1: PRISMA flow diagram summarizing the identification, screening, and selection process

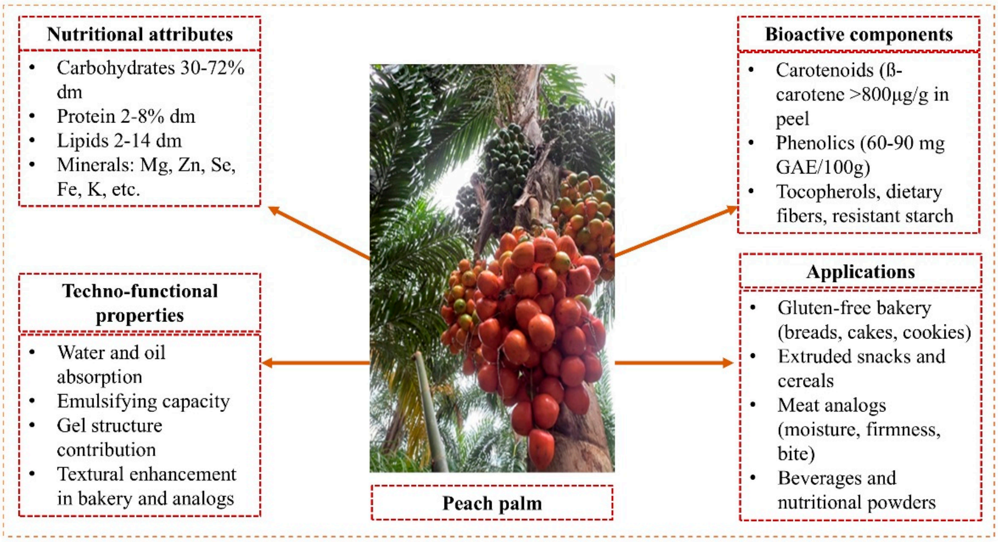

Figure S2: Integrative summary of the nutritional, bioactive, techno-functional, and application potential of peach palm (Bactris gasipaes) in plant-based food systems.
